# Outcome in patients with open abdomen treatment for peritonitis: a multidomain approach outperforms single domain predictions

**DOI:** 10.1007/s10877-021-00743-8

**Published:** 2021-07-10

**Authors:** Sven Petersen, Markus Huber, Federico Storni, Gero Puhl, Alice Deder, Axel Prause, Joerg C. Schefold, Dietrich Doll, Patrick Schober, Markus M. Luedi

**Affiliations:** 1 Department of General and Visceral Surgery, Asklepios Hospital Altona, Hamburg, Germany; 2 Department of Anaesthesiology and Pain Medicine, Inselspital, Bern University Hospital, University of Bern, Freiburgstrasse, 3010 Bern, Switzerland; 3 Department of Visceral Surgery and Medicine, Inselspital, Bern University Hospital, University of Bern, Bern, Switzerland; 4 Department of Anaesthesiology, Asklepios Hospital Altona, Hamburg, Germany; 5 Department of Intensive Care Medicine, Inselspital, Bern University Hospital, University of Bern, Bern, Switzerland; 6 Department of Procto-Surgery, St. Marien-Krankenhaus Vechta, Vechta, Germany; 7 Department of Anaesthesiology, Amsterdam University Medical Centres, Vrije Universiteit Amsterdam, Amsterdam, the Netherlands

**Keywords:** Decision support, Peritonitis, Open abdomen, Mortality, SAPS-II score, MPI score

## Abstract

Numerous patient-related clinical parameters and treatment-specific variables have been identified as causing or contributing to the severity of peritonitis. We postulated that a combination of clinical and surgical markers and scoring systems would outperform each of these predictors in isolation. To investigate this hypothesis, we developed a multivariable model to examine whether survival outcome can reliably be predicted in peritonitis patients treated with open abdomen. This single-center retrospective analysis used univariable and multivariable logistic regression modeling in combination with repeated random sub-sampling validation to examine the predictive capabilities of domain-specific predictors (i.e., demography, physiology, surgery). We analyzed data of 1,351 consecutive adult patients (55.7% male) who underwent open abdominal surgery in the study period (January 1998 to December 2018). Core variables included demographics, clinical scores, surgical indices and indicators of organ dysfunction, peritonitis index, incision type, fascia closure, wound healing, and fascial dehiscence. Postoperative complications were also added when available. A multidomain peritonitis prediction model (MPPM) was constructed to bridge the mortality predictions from individual domains (demographic, physiological and surgical). The MPPM is based on data of n = 597 patients, features high predictive capabilities (area under the receiver operating curve: 0.87 (0.85 to 0.90, 95% CI)) and is well calibrated. The surgical predictor “skin closure” was found to be the most important predictor of survival in our cohort, closely followed by the two physiological predictors SAPS-II and MPI. Marginal effects plots highlight the effect of individual outcomes on the prediction of survival outcome in patients undergoing staged laparotomies for treatment of peritonitis. Although most single indices exhibited moderate performance, we observed that the predictive performance was markedly increased when an integrative prediction model was applied. Our proposed MPPM integrative prediction model may outperform the predictive power of current models.

## Introduction

Primary and secondary infectious peritonitis pose major challenges for clinicians globally [1, 2] . Despite extensive research and aggressive surgical management of source control–such as open abdominal clinical strategies with repeated abdominal lavage or interventions–the prognosis of generalized peritonitis remains poor, with mortality rates of up to 60% [3] . There is some evidence of favorable outcomes when using staged relaparotomies and repeated lavage in selected cohorts [1, 4, 5] . This approach is now accepted as a standard procedure for the septic abdomen [6, 7].

Patients usually undergo explorative laparotomy if peritonitis is suspected. Following meticulous exploration of the abdomen, thorough cleansing with copious fluids, and surgical repair of lesions, the abdominal cavity is left open and the small bowel is protected with an intestine bag. To minimize abdominal wall retraction during open abdomen treatment, a mesh is sutured into the dorsal aspect of the rectus muscle.

Despite considerable clinical and scientific efforts, mortality remains high in these patients, and predictive scores are warranted to change the clinical approach from “reaction to deterioration” towards a more proactive “anticipation of deterioration”. [8] Bosscha et al. previously stated that early and reliable classification of intra-abdominal sepsis is essential. This could further be useful to select patients for aggressive surgical techniques and to evaluate and compare the results of different clinical treatment regimens [9] . Bosscha et al. demonstrated an association of the Mannheim Peritonitis Index (MPI) and the Acute Physiology And Chronic Health Evaluation (APACHE) II in a cohort of peritonitis patients [9] . In light of the study design and the focus on chronic concomitant diseases, the best resource allocation and acute surgical management in these patients remains controversial [10] . Another group assessed the feasibility of predicting mortality with a country-specific calibrated Simplified Acute Physiology Score (SAPS) II in intensive care (ICU) patients, and showed that overall mortality is overestimated when the SAPS II is used [11] . We hypothesized that an integrative approach may be useful to improve prediction modeling in patients with peritonitis, and could include demographics, typical physiological scoring systems (SAPS II and MPI) as well as a specific clinical course.

The need for advanced prediction models might lead us to a better understanding of the variables that affect this clinical challenge. Therefore, we investigated the utility of a newly designed multivariable approach that deliberately employs laborartory results and clinical and surgical indices. This was done in an effort to best reflect the clinical challenges facing a large cohort of peritonitis patients undergoing open abdomen and staged lavage treatment. We combined typical clinical scores with surgical parameters, as this might best reflect the decision-making performed by health care professionals.

## Methods

The study was performed in adherence to the principles in the Declaration of Helsinki, and approved by the Hamburg Medical Association (#WF072/20) as the responsible institutional review board/human ethics board. The need for individual patients’ or legal surrogates’ or parents’ or legal guardians’ written informed consent was deemed unnecessary, given the retrospective nature of the data analysis.

### Patients

Data of 1,351 adult patients treated for peritonitis in the Department of General and Visceral Surgery of the Asklepios Hospital Altona, Hamburg, Germany, were analyzed retrospectively. All adult patients were treated in the unit ICU and had undergone open abdomen treatment and staged lavage during the study interval (January 1998 to December 2018). For “transparent reporting of a multivariable prediction model for individual prognosis or diagnosis” we followed the Equator TRIPOD statement [12].

### Measurements

Age at admission, Mannheim Peritonitis Index (MPI), number of staged lavages, duration of mechanical ventilation (in hours), incision type (median vs. transverse) during staged lavage, fascia closure at the end of the staged lavage, presence of a wound-healing disorder, fascial dehiscence, postoperative complications, and mortality were documented.

SAPS-II scores [13–15] were available for the period 1998 to 2018 and MPI scores from 2008 to 2018. SAPS-II scores were lower during the years 1998 to 2007 (median 40.0 and interquartile range [32.0–52.0]) than during the period 2008–2018 (46.0 [36.0–57.0]), for which both physiological scores were available (p < 0.001). This possible source of bias in our analysis of mortality prediction is discussed within the context of the limitations of this study. A full data availability atable is presented in the Supplementary Material.

The following standard surgical procedures applied: type of incision was based on the incision previously performed. When no previous abdominal incisions or laparoscopic trocar incisions were recorded or observed, a transverse incision was used. All four quadrants were inspected and thoroughly cleaned with copious fluid. After ensuring that no infectious pockets were left undrained, the abdominal cavity was left open, the small bowel was covered with negative pressure (Vi-Drape® intestine bag, Cardinal Health GmbH, 22,848 Norderstedt, Germany), and Parietex® mesh (Medtronic GmbH, 40,670 Meerbusch, Germany) was sutured into the dorsal aspect of the rectus muscle.

Wound-healing disorders (WHD) were defined as any evidence of cutaneous wound infection or need for reopening of a skin wound, with or without bacteria detected in microbiological swabs. Any evidence of reopening of the fascia after the closure of staged lavage was defined as fascial dehiscence. Data on the severity of the disease, as determined by the SAPS-II score, were collected upon admission to the intensive care unit. The surgical results were determined retrospectively.

### Statistical analyses

Continuous variables were expressed as mean and standard deviation when normally distributed, based on a Shapiro–Wilk test of normality and visual inspection of Q-Q plots, and as median and interquartile range (IQR) otherwise [16] . Differences in a continuous outcome between two groups were assessed with Student’s T-test in case of normally distributed outcomes and with a Mann–Whitney Test otherwise. Proportions are presented as numbers and percentages, and tests of association of two groups with a binary outcome were performed using a chi-square test.

To assess the ability of the various demographic, physiological and surgical variables to predict the binary survival outcome, we first computed univariable logistic regression for each predictor. Second, those predictors associated with the mortality outcome and with sufficient observations were selected and combined in domain-specific multivariable logistic regression models (demographics, physiological, surgical) to examine the individual prediction skill of each domain. Finally, all predictors were combined in a multivariable logistic regression model [17] . Goodness of fit of these models was assessed with the Hosmer–Lemeshow test as well as with calibration plots, and overall model performance was quantified using the Brier Score [18, 19] . The discriminative ability of each logistic regression model was computed with concordance statistics/the area under the receiver operating curve (AUROC) [20, 21] . Predictor importance in the multi-domain prediction model was assessed with two methods: (i) the absolute value of the t-statistic for each model parameter and (ii) dominance analysis [22, 23] . In terms of missing data, we followed a complete case analysis and omitted missing values for each regression model.

In order to (i) internally validate the models’ ability to discriminate between the survival outcome (as expressed with the AUROC values for each regression model) and (ii) to compare the predictive skill across single predictor models, domain-specific models and the multidomain model, we employed a repeated random sub-sampling validation for each regression model. The following steps were repeated a thousand times for each regression model: (1) The available data was randomly divided into a training set (containing 65% of the available data) and the logistic regression model was fit using this training data. (2) To categorize the model prediction probabilities into the binary survival outcome categories (survived, died), an optimal cutoff value for the predicted probabilities was computed according to the Youden Index. (3) The fitted regression model—in combination with the optimal cutoff value—was subsequently used to predict the individual binary outcomes of the validation set (containing the remaining 35% of the data). (4) The following indicators of prediction performance were computed for the validation set: balanced accuracy, sensitivity, specificity, positive predictive value, negative predictive value and the diagnostic odds ratio [24] . Overall, this validation procedure resulted in a distribution with 1,000 samples for the indicators of prediction performance which were depicted with box plots.

To determine the sample size required for the logistic regression models, we followed the method of Peduzzi et al. 1996: [25] assuming 10 possible covariates in the full multi-domain regression model and a mortality rate of 20%, we calculated 500 as the minimum number of patients. A p < 0.05 was considered statistically significant. All statistical analyses were computed with R (R version 4.0.2; R Core Team 2020). Calibration plots were computed with the package givitiR [26].

## Results

### Demographics and clinical outcome

Data of 1,351 consecutive patients undergoing staged laparotomies were analyzed. Table 1 describes the patients’ demographic, physiological and surgery-related variables and compares the variable distribution in surviving and deceased patients. In-hospital survivors were younger (median 64.0 years and interquartile range [52.0–73.0] years than non-survivors (73.0 [63.0–78.0] years, p < 0.001). They had lower SAPS-II scores (40.0 [33.0–50.0] versus 56.0 [45.0–66.0], p < 0.001) and lower MPI scores (19.0 [12.2–26.0] versus 26.0 [16.0–32.2], p < 0.001). And they required less time on mechanical ventilation (140 [63.0–352] hours versus 306 [74.0–538] hours, p < 0.001). In terms of surgical procedures, fascial and skin closure and evidence of wound healing disorder were associated with clinical outcome (p < 0.001).

**Table 1:** Demographic, physiological and surgery-related variables in the cohort of patients undergoing staged laparotomies for peritonitis

	All patients (*N* = *1351)*	Survived (*N* = *1082)*	Died (*N* = *269)*	*P*
*Demographics*				
Sex (male)	750 (55.7%)	587 (54.4%)	163 (60.8%)	0.068
Age (years)	66.0 (54.0–75.0)	64.0 (52.0–73.0)	73.0 (63.0–78.0)	< 0.001
Height (cm)	170 (163–178)	170 (163–178)	170 (160–176)	0.258
Weight (kg)	75.0 (65.0–85.2)	75.0 (65.0–85.5)	76.0 (64.0–85.0)	0.835
BMI (kg/m^2^)	25.1 (22.6–28.7)	25.1 (22.7–28.4)	25.5 (22.5–29.3)	0.694
*Physiology*				
SAPS-II Score	43.0 (34.0–54.0)	40.0 (33.0–50.0)	56.0 (45.0–66.0)	< 0.001
Mannheimer Peritonitis Index (MPI)	21.0 (14.0–28.0)	19.0 (12.2–26.0)	26.0 (16.0–32.2)	< 0.001
*Number of lavages*				*0.727*
1	709 (52.5%)	576 (53.2%)	133 (49.4%)	
2	270 (20.0%)	209 (19.3%)	61 (22.7%)	
3	142 (10.5%)	111 (10.3%)	31 (11.5%)	
4	85 (6.29%)	71 (6.56%)	14 (5.20%)	
5	38 (2.81%)	30 (2.77%)	8 (2.97%)	
> 5	107 (7.92%)	85 (7.86%)	22 (8.18%)	
Days in ICU	10.0 (4.00–21.0)	9.00 (4.00–20.0)	13.0 (3.00–24.0)	0.019
Hours of ventilation	157 (64.0–402)	140 (63.0–352)	306 (74.0–538)	< 0.001
Hours of hemofiltration	154 (63.5–306)	164 (96.2–296)	133 (30.0–316)	0.057
*Surgery*				
Index operation				0.768
Median laparotomy	146 (24.7%)	104 (23.9%)	42 (26.8%)	
Transverse laparotomy	374 (63.2%)	277 (63.7%)	97 (61.8%)	
Others	72 (12.2%)	54 (12.4%)	18 (11.5%)	
*Open abdomen treatment*				0.906
Median	158 (26.8%)	117 (27.0%)	41 (26.5%)	
Transverse	416 (70.6%)	305 (70.3%)	111 (71.6%)	
Others	15 (2.55%)	12 (2.76%)	3 (1.94%)	
*Fascia closure*				< 0.001
No	55 (9.20%)	4 (0.91%)	51 (31.9%)	
Yes	543 (90.8%)	434 (99.1%)	109 (68.1%)	
*Skin closure*				< 0.001
No	57 (9.53%)	6 (1.37%)	51 (31.9%)	
Yes	541 (90.5%)	432 (98.6%)	109 (68.1%)	
*Wound healing disorder*				0.002
No	455 (76.0%)	318 (72.6%)	137 (85.1%)	
Yes	144 (24.0%)	120 (27.4%)	24 (14.9%)	
*Fascia complication*				0.861
No	558 (93.2%)	409 (93.4%)	149 (92.5%)	
Yes	41 (6.84%)	29 (6.62%)	12 (7.45%)	
*Vacuum treatment*				0.374
No	553 (92.5%)	402 (91.8%)	151 (94.4%)	
Yes	45 (7.53%)	36 (8.22%)	9 (5.62%)	

### Domain-specific prediction models

We next considered the association between individuals’ demographic, physiological and surgical variables and the binary survival outcome (Table 2). Here, univariable logistic regression reveals significant associations between mortality and patient age, SAPS-II and MPI scores, the number of days in the ICU, as well as for wound complications and surgical management (skin and fascial closure). The area under the curve (AUROC) is shown as a measure of the predictive value of the univariable model. Here the SAPS-II regression model provides the highest AUROC value of 0.75 (0.72–0.79, 95% CI) relative to the other variables, with AUROC values in the range of 0.51 (0.47–0.55) (BMI) to 0.66 (0.63–0.70) (Age).

**Table 2:** Univariable logistic regression models for the binary survival outcome

	OR	95% CI	*P*	AUROC	Brier	Hoslem	McFadden
Age	1.04	1.03,1.05	< 0.001	0.66 (0.63–0.69)	0.15	0.983	0.05
Sex [Male]	1.30	0.99,1.71	0.059	0.53 (0.50–0.56)	0.16	1.000	0.00
BMI	1.00	0.98,1.03	0.72	0.51 (0.46–0.55)	0.16	0.680	0.00
SAPS-II	1.06	1.05,1.07	< 0.001	0.75 (0.72–0.79)	0.14	0.058	0.14
MPI	1.06	1.04,1.08	< 0.001	0.65 (0.60–0.70)	0.18	0.015	0.05
Days in ICU	1.01	1.00,1.01	0.019	0.55 (0.51–0.59)	0.16	0.000	0.00
Hemofiltration [Yes]	8.72	6.22,12.29	< 0.001	0.66 (0.63–0.69)	0.14	1.000	0.12
WHD [Yes]	0.46	0.28,0.74	0.002	0.56 (0.53–0.60)	0.19	1.000	0.02
Index Operation				0.52 (0.47–0.56)	0.19	1.000	0.00
Median	ref						
Transverse	0.87	0.57,1.34	0.512				
Others	0.83	0.43,1.55	0.558				
OAT				0.51 (0.47–0.55)	0.19	1.000	0.00
Median	Ref						
Transverse	1.04	0.69,1.59	0.859				
Others	0.71	0.16,2.38	0.615				
Fascia closure [Yes]	0.02	0.01,0.05	< 0.001	0.65 (0.62–0.69)	0.15	1.000	0.17
Skin Closure [Yes]	0.03	0.01,0.07	< 0.001	0.65 (0.62–0.69)	0.15	1.000	0.16

*OR*  odds ratio, *CI*  confidence interval, *OAT* open abdomen treatment, *WHD* wound healing disorders

**Fig. 1:**
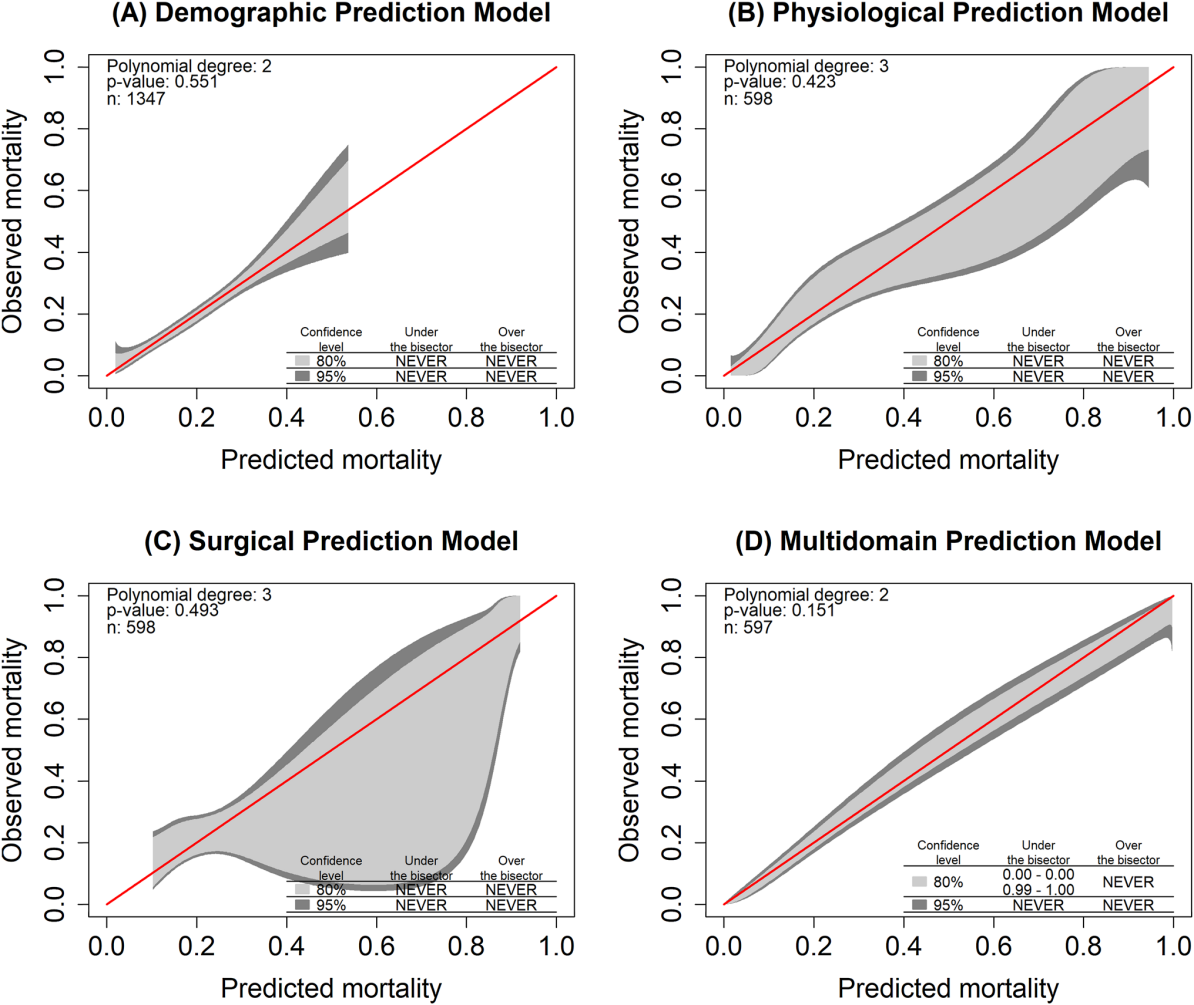
Calibration plots of predicted mortality versus observed mortality. Calibration plots of predicted mortality versus observed mortality using demographic predictors (age and sex of the patients; panel **A**), physiological predictors (SAPS-II and MPI scores; panel **B**), surgical predictors (wound healing disorders and skin closure; panel **C**). Panel **D** illustrates the calibration of the *multidomain peritonitis prediction model,* which includes the predictors from all three domains. The diagonal red lines denote a 1:1 relationship between predicted and observed mortality

To examine the predictive capabilities of the demographic, physiological and surgical domain, we grouped the predictors into domain-specific multivariable logistic regression models (Table 3). Calibration plots for these three models of predicted mortality versus observed mortality in our cohort are shown in Fig. 1. The demographic prediction model is well calibrated, whereas the physiological prediction underestimates the mortality for low probabilities on the one hand and overestimates high mortality probabilities on the other hand. The surgical prediction model generally overestimates the observed mortality in the cohort.

**Table 3:** Domain-specific multivariable logistic regression model for the binary survival “outcome”

	OR	95% CI	p-value
*Demographic prediction model*^a^			
Age	1.04	1.03, 1.06	** < 0.001**
Sex [male]	1.45	1.09, 1.92	**0.010**
*Physiological prediction model*^b^			
SAPS-II Score	1.06	1.05, 1.08	** < 0.001**
MPI Score	1.05	1.03, 1.07	** < 0.001**
Days in ICU	0.99	0.98, 1.01	0.3
*Surgical prediction model*^c^			
Wound healing disorders [Yes]	0.38	0.21, 0.65	< 0.001
Skin closure [Yes]	0.03	0.01, 0.06	< 0.001

*OR* odds ratio, *CI* confidence interval

^a^N = 1347, Brier-Score 0.15, AUROC 0.67 (0.63–0.7), Hosmer–Lemeshow Goodness of Fit Test 0.99

^b^N = 598, Brier-Score 0.16, AUROC 0.77 (0.73–0.82), Hosmer–Lemeshow Goodness of Fit Test 0.76

^c^N = 598, Brier-Score 0.15, AUROC 0.69 (0.65–0.74), Hosmer–Lemeshow Goodness of Fit Test 1.00

We found that patient age was the strongest predictor of the demographic variables, with an odds ratio (OR) of 1.04 (1.02–1.06, 95% CI). A unit increase in the SAPS-II score and MPI score increases the odds of mortality by 6% (5%–8%) and 5% (3%–7%), respectively. The odds of survival strongly increase in cases of successful skin closure, with an odds ratio of 0.03 (0.01–0.06). In the context of mortality prediction, the demographic model shows a moderate AUROC value of 0.67 (0.63–0.70), with a similar AUROC of 0.69 (0.65–0.74) for the surgical predictors. The physiological prediction model shows the highest AUROC of 0.77 (0.73–0.82) of the domain-specific prediction models.

### The integrative prediction models

**Fig. 2:**
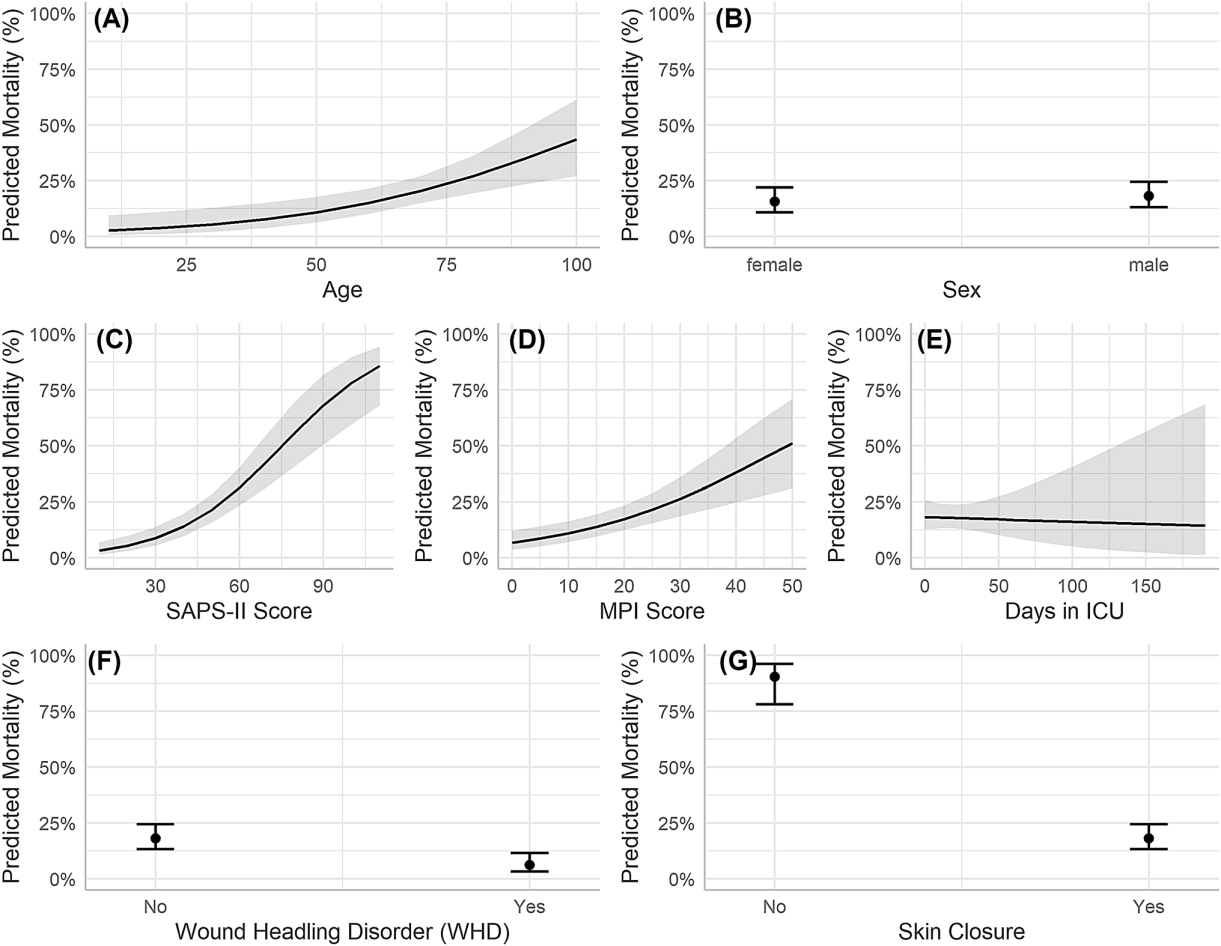
Marginal effects plots of the *multidomain peritonitis prediction model.* Shaded bands and error bars denote the 95% confidence interval. **A**, **B** demographic predictors, **C**-**E** physiological predictors and **F**-**G** surgical predictors. Only one predictor is varied in each panel while the other predictors are held constant: here, the predictor-specific predictions are adjusted for a 66 year old male patient with SAPS-II and MPI scores of 46 and 21, respectively, 21 days at ICU with no wound healing disorders and successful skin closure. Note that changing these adjustment values would result only in a vertical shift the outcome predictions – the shape of the curves as well as the prediction differences between categories would remain the same

A final prediction model—the multidomain peritonitis prediction model (MPPM)—was constructed (Table 4). The model is built upon the clinical observation that an integrative data analysis might best indicate clinical outcomes. The MPPM is based on data of n = 597 patients, features a high AUROC value of 0.87 (0.85–0.90) and a Brier Score of 0.12, and is well calibrated (Fig. 1D). Figure 2 illustrates the marginal effects for each predictor, and highlights that mortality steadily increases for older patients and higher SAPS-II and MPI scores. For example, a SAPS-II score of 80 predicts a survival probability of 56% (41%–70%, 95% CI), holding the other predictors at the values referenced in Fig. 2 constant. Skin closure at the end of surgery is a powerful predictor of survival: in patients in whom skin closure cannot be achieved, mortality is predicted to be 90% (78%–96%) when the other predictors are held constant.

**Table 4:** The final multivariable logistic regression models for the binary survival outcome (*multidomain peritonitis prediction model*)

Multidomain peritonitis prediction model^a^	OR	95% CI	p-value
Age	1.04	1.02, 1.06	** < 0.001**
Sex [male]	1.19	0.74, 1.95	0.47
SAPS-II Score	1.05	1.04, 1.07	** < 0.001**
MPI Score	1.06	1.03, 1.08	** < 0.001**
Days in ICU	1.00	0.98, 1.01	0.8
Wound healing disorders (WHD) [Yes]	0.29	0.15, 0.54	< 0.001
Skin closure [Yes]	0.02	0.01, 0.06	< 0.001

*OR*  odds ratio, *CI* confidence interval

^a^N = 597, Brier-Score 0.12, AUROC 0.87 (0.85–0.90), Hosmer–Lemeshow Goodness of Fit Test 0.605

Supplemental Fig. 2 illustrates the relative importance of the individual predictors in the integrative MPPM model. Estimates of the relative importance based on the absolute value of the t-statistic in the prediction model are shown in Panel A, whereas dominance analysis was employed to compute the estimates of relative importance in Panel B. The latter method systematically examines all possible subsets of the model predictors and evaluates the additional contribution of a particular predictor to a measure of model fit (in our case McFadden’s R^2^). The two independent methods agree in their overall ranking of variable importance.

The physiological predictors SAPS-II score and MPI score are slightly less important than skin closure.

The demographic predictors appear to be only marginally important relative to the surgical and physiological predictors.

### Comparison of domain-specific and integrative prediction models

**Fig. 3:**
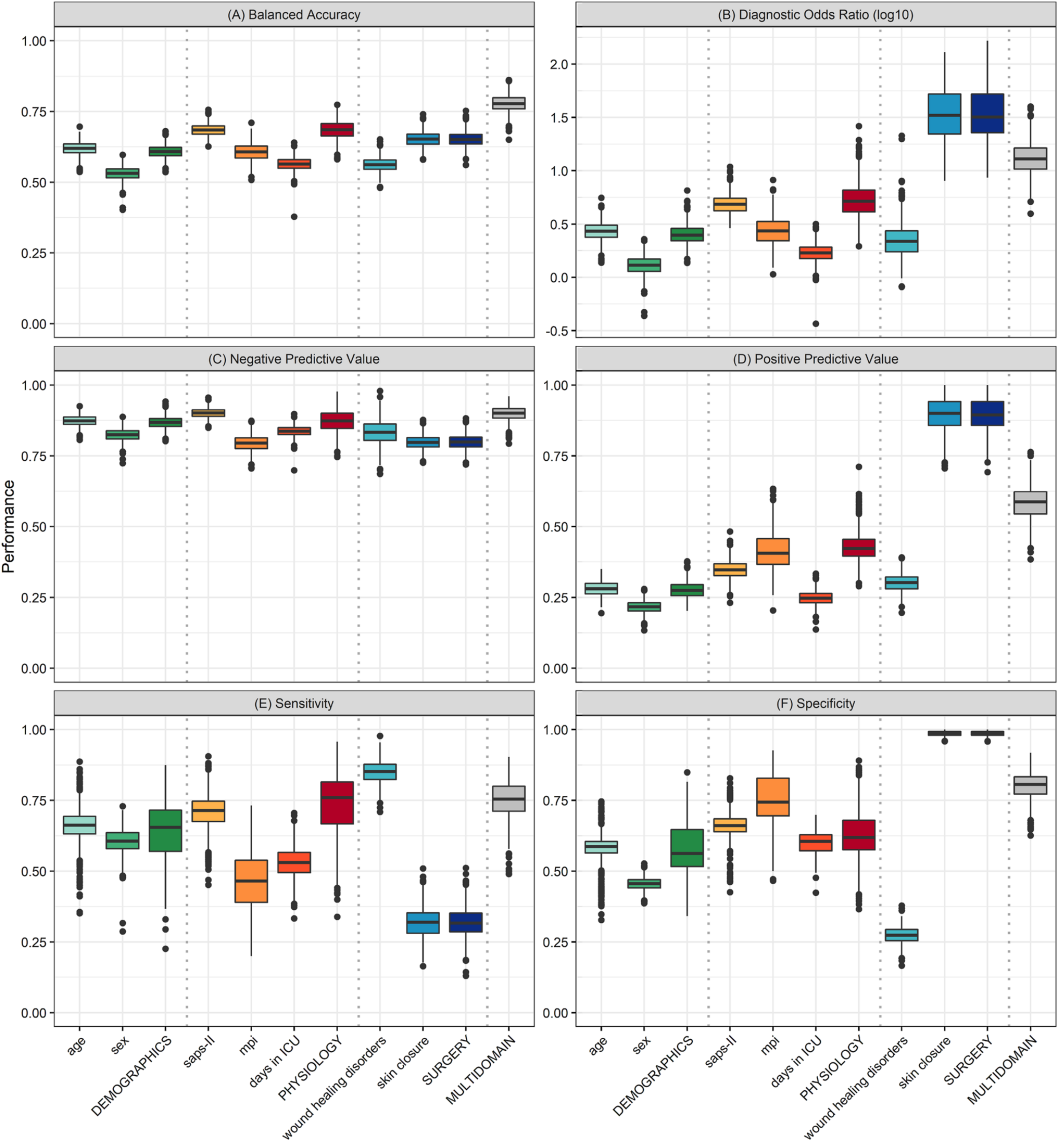
Diagnostic performance of single predictor models, domain-specific models and the *multidomain peritonitis prediction model* in predicting the survival outcome in patients with open abdomen treatment for peritonitis. A repeated random sub-sampling validation was used to compute distributions of quantitative indicators (balanced accuracy, log diagnostic odds ratio, negative predictive value, positive predictive value, sensitivity and specificity). Box plots illustrate the median and interquartile ranges of these distribution. Capitalized predictors denote logistic regression models including all predictors of a particular domain, i.e., the model DEMOGRAPHICS includes the age of the patient and sex as predictors

To conclude, we compared the predictive ability of the single predictor models and the three domain-specific models (demographic, physiological, surgical) with the integrative multidomain peritonitis prediction model within a repeated random sub-sampling validation framework. Figure 3 shows the median and interquartile ranges for a suite of performance indicators in the 1,000 random sub-sampling ensemble. Note that the regression coefficients used for the mortality prediction probabilities and the optimal cutoff for distinguishing between survival and death were solely based on the data of random training sets. The MPPM model features the overall highest balanced accuracy, with a median of 78% (76%–80%, interquartile range). The surgical-domain model demonstrates a higher diagnostic odds ratio (median 34; IQR: 24–62) than the multidomain model (median 13; IQR: 10–16). It is characterized by a low proportion of correctly identified positives (sensitivity) but a high proportion of correctly identified negatives (specificity).

While individual domain-specific models show capabilities similar to those of the MPPM for some indicators, such as the physiological model for sensitivity, Fig. 3 illustrates the key finding of our study—that combining the predictors of various domains increases the overall ability to predict the binary survival outcome in patients undergoing staged laparotomies for peritonitis treatment (see Fig. 1).

## Discussion

Prediction modeling seems of great clinical importance in the clinical scenario investigated. Current predictions mostly rely on single-index analyses. We demonstrate that integrative modeling using available information about demographics, disease severity, physiological parameters, and medical interventions can outperform previous prediction models, highlighting the importance of our integrative (MPPM model) approach.

Concerning peritonitis treatment, there has been ongoing discussion for years as to whether open abdomen treatment is justified, or whether a so-called “second look on demand” makes more sense. Although this is not the main focus of this analysis, the data presented here show a relatively low mortality rate compared to publications showing the results of second-look on-demand patients [5, 31, 32] . Cocollini from the “International Register of open abdomen” concluded that temporary abdominal closure remains reliable and safe as a treatment for severely injured and acute care surgery patients [33]. For peritonitis, the second major endpoint in case of an open abdomen is closure at the end of open treatment. In recent years, multiple working groups have put considerable effort into evaluating vacuum-assisted therapy as a treatment option for peritonitis in order to improve the closure rate [34, 35] . The benefits of vacuum-assisted therapy are that the effort needed for repeated lavage treatments is minimized and the rate of patients with successful abdominal wall closure is higher. In our analysis the observed closure rate using open abdomen treatment was 87%, which is relatively high compared to studies using vacuum-assisted options [36–38] . Either therapy is futile, though, if the predicted outcome is bleak.

Outcome prediction is typically performed using single clinical and surgical markers and isolated scoring systems such as the SAPS II score. We showed that a combination of those dimensions outperforms predictions based on single indices. This was possible because the data analysis was based on a large single-center group of patients with peritonitis. The patients’ individual factors were collected prospectively for the SAPS-II score, with further treatment-specific factors being added in a retrospective analysis.

The major advantage of this data analysis is its consistent cohort, with open abdomen treatment being performed uniformly over two decades. Intensive care strategies have also remained unchanged. As demonstrated in Fig. 1, there are only minor changes in the SAPS-II scoring evaluated over 20 years.

### Limitations

Our work has several important limitations that deserve discussion. First, data were assessed retrospectively, with all inherent limitations driven by study design. While data were consistently documented in a timely manner following OR procedures, recall bias could theoretically apply. Importantly, the MPI was calculated from findings during surgery [27] . While this scoring system leaves room for interpretation and is logically limited in terms of power, the MPI was shown to be an accepted tool for mortality prediction [28] . It should be emphasized that only in-hospital mortality was analyzed.

Second, regardless of the power of our proposed prediction model, no single clinical scoring system should be a substitute for clinical decision-making. Nevertheless, although we regard it as a strength of our model that a multi-variable approach deliberately includes important clinical variables, clinical decision-making should not solely be based on even such sophisticated models.

Using the SAPS-II score, the majority of vital signs—including oxygenation, renal function and results from blood samples—were included at the time of admission to the ICU [15] . In addition, the data availability changed in the middle of the observation period, when additional physiological (MPI score) and surgical predictors (i.e., wound-healing disorder) became available. We thus note that the comparison of univariable prediction models as well as the comparison of domain-specific models (for example, the physiological prediction model versus the surgical prediction model) are not based on the same patients but rather on different subsamples of the entire cohort.

## Conclusion

Currently, prediction modeling mainly relies on single-index (or combination-index) analyses. With the *multidomain peritonitis prediction model* we demonstrate that integrative modeling using available information such as demographics, disease severity, physiological parameters, and medical interventions outperforms previous models. In the case of a severely compromised patient with peritonitis, our model suggests that the predictive power is best when all predictive parameters from the performance status are combined. This could lead to more reliable outcome prediction, and reflects the great importance of the interdisciplinary combination of surgical, laboratory, and clinical expertise, leading to improved decision-making in experienced physicians.
